# The effect of patient, provider and financing regulations on the intensity of ambulatory physical therapy episodes: a multilevel analysis based on routinely available data

**DOI:** 10.1186/s12913-015-0686-6

**Published:** 2015-02-07

**Authors:** Patricia Halfon, Yves Eggli, Yves Morel, Patrick Taffé

**Affiliations:** Institute of Social and Preventive Medicine (IUMSP), University Hospital Center and Faculty of Biology and Medicine, Biopole 2, Route de la Corniche 10, 1010 Lausanne, Switzerland; Institute of Health Economics and Management, University Hospital Center and University of Lausanne, Route de Chavannes 31, 1015 Lausanne, Switzerland

**Keywords:** Physical therapy, Episodes of care, Explained variation, Multilevel models, Crossed random effects

## Abstract

**Background:**

Many studies have found considerable variations in the resource intensity of physical therapy episodes. Although they have identified several patient- and provider-related factors, few studies have examined their relative explanatory power. We sought to quantify the contribution of patients and providers to these differences and examine how effective Swiss regulations are (nine-session ceiling per prescription and bonus for first treatments).

**Methods:**

Our sample consisted of 87,866 first physical therapy episodes performed by 3,365 physiotherapists based on referrals by 6,131 physicians. We modeled the number of visits per episode using a multilevel log linear regression with crossed random effects for physiotherapists and physicians and with fixed effects for cantons. The three-level explanatory variables were patient, physiotherapist and physician characteristics.

**Results:**

The median number of sessions was nine (interquartile range 6–13). Physical therapy use increased with age, women, higher health care costs, lower deductibles, surgery and specific conditions. Use rose with the share of nine-session episodes among physiotherapists or physicians, but fell with the share of new treatments. Geographical area had no influence. Most of the variance was explained at the patient level, but the available factors explained only 4% thereof. Physiotherapists and physicians explained only 6% and 5% respectively of the variance, although the available factors explained most of this variance. Regulations were the most powerful factors.

**Conclusion:**

Against the backdrop of abundant physical therapy supply, Swiss financial regulations did not restrict utilization. Given that patient-related factors explained most of the variance, this group should be subject to closer scrutiny. Moreover, further research is needed on the determinants of patient demand.

**Electronic supplementary material:**

The online version of this article (doi:10.1186/s12913-015-0686-6) contains supplementary material, which is available to authorized users.

## Background

Interest in physical therapy utilization has grown in recent decades. In the United States, national inflation-adjusted expenditures for spine-related physical therapy have increased by 78% between 1997 and 2005, which is more than the 65% rise in total estimated care expenditures for the condition [1]. Several authors tried to understand if physical therapy is used properly in comparing its utilization in different settings. They found variations across countries in terms of the intensity of physical therapy per treatment episode [2]. There is a variety of acute or chronic conditions justifying physical therapy, one of the main complaint being back pain [3,4]. The treatment of this condition required an average of five sessions in United Kingdom, while the US average was 11 [5,6].

Variations in physical therapy use were analyzed based on the propensity to start physical therapy [3,7-10], the number of sessions per treatment episode [9,11-13] and costs [13,14]. Several studies have analyzed the determinants of the intensity of physical therapy use, highlighting the role of both health-related factors like age, gender, site and severity of the condition and poor health [1,8,11-15] and others, such as insurance status, education level, income and urban residence [1,12,13,15]. However, even after most of these factors are taken into account, the treatment intensity can still vary substantially between patients [12,16]. A Dutch study suggested that there was an overuse of physical therapy for lower back pain; patients with acute complaints, in particular, attended a much higher number of sessions than recommended. Moreover, only a minority of patients with lower back pain was treated according to national physical therapy guidelines [17]. The introduction of professional guidelines coupled with national volume policy led to a decline in the number of physical therapy appointments and an increase in the use of evidence-based methods to treat lower back pain [18].

We were unable to find any analyses of the relative role of patients, physicians, physiotherapists and financing regulations on the volume of physical therapy used. Switzerland is an interesting setting because there is a high density of physiotherapists, as well as a number of control mechanisms in place (deductibles, ceiling per prescription, and higher fees for first session). The density of physiotherapists is estimated at 1/700 inhabitants, which is twice the average density observed in Europe [19]. Roughly half of physiotherapists are in private practice [20]. In 2006, physical therapy was responsible for 7.5% of allied care costs and 1.3% of ambulatory health care services costs (including auxiliary services such as transport, radiology and laboratory) [21].

The primary objective of our study was to ascertain the extent to which the volume of physical therapy per treatment episode depended on patient characteristics and/or on physician and physiotherapist behaviors, and whether Swiss regulations were effective. To this end, we used routinely collected data on a large national representative sample of insured, which enabled us to analyze a broad range of conditions.

## Methods

### Setting

In Switzerland, the compulsory health insurance system requires a physician referral for physical therapy treatment. The insured pay out 100% of the costs until the deductible is reached (100 to 2500 Swiss francs depending on the policy chosen by the insured) and continue to pay 10% of additional expenses up to a fixed ceiling. Cantons may restrict the opening of new private physical therapy practices, although this has yet to happen. Patients are free to choose their therapist, who are paid according to a set price per session and depending on the type of service they provide (general physical therapy, complex session, horse therapy, manual lymphatic drainage). They may also charge additional fees for special services (including swimming pool and home treatment) [22].

For many years regulations have placed two main constraints on physical therapy use. First, there is a maximum of nine sessions per physician prescription; a new prescription is required to repeat the treatment (maximum of 9 sessions), but there is no limitation on the number of prescriptions the insured can have if the same condition requires subsequent treatment. Second, the fee for the first session of a new treatment is higher. Regarding long-term treatments (>36 sessions), the insurer, after consulting with the referring physician, is entitled to demand medical checks, impose time limits on treatment and restrict the type of physical therapy. In spite of physician gatekeeping, physiotherapists are free to choose the therapy they administer and are expected to limit the number of sessions to patient needs and health objectives regardless of the original physician prescription. Since the end of the study period, neither the organization of the physiotherapy profession nor the regulations have changed.

### Studied population

We undertook an observational study based on routine data from four Swiss health insurers, covering 2.02 million insured enrollees (27% of the Swiss population), of whom 1.68 million were followed in 2005 and 2006. Data included all claims for ambulatory services, dispensed drugs and hospitalizations. Although cantonal distribution differed markedly between this population and the general Swiss population - reflecting the preferential coverage that the four health insurance companies offered in certain cantons - the age, gender and deductible distribution were similar [23]. We retained all the insured, followed until 31 December 2006, who had their first physical therapy session in 2005. We included all sessions delivered by licensed physiotherapists and outpatient hospitals. Insurers’ data are not publicly available and were supplied for the sole purpose of the present research project, which received Swiss Federal Office of Public Health support [24]. Due to the fact that physiotherapists are paid directly by insurers, we expect few inaccuracies in the data collected for billing purposes. Physicians and physiotherapists had their own anonymous identification number. All data on patients and care providers were also anonymous and contained no information, such as date of birth or ZIP code, which would make it possible to identify individuals [25].

### Conceptual model

**Figure 1:**
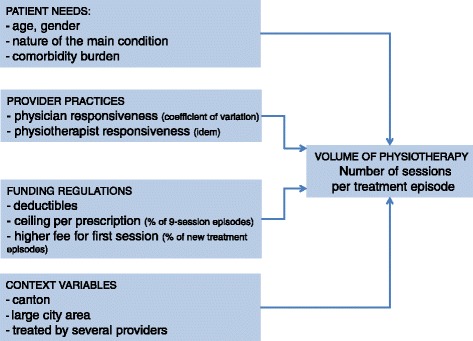
**Conceptual model.**

There are four determinants that could possibly influence the volume of physical therapy (Figure 1): patient needs, provider practices (physicians and physiotherapists), incentive and dissuasive funding regulations and context variables.

#### Dependent variable

**Figure 2:**
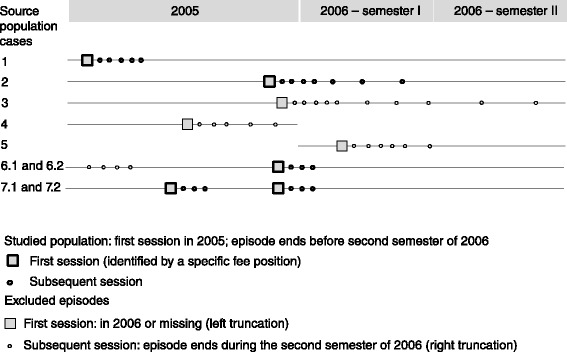
**Definition of studied episodes.**

The outcome variable was the number of physical therapy sessions per treatment episode. An episode began with the first session of a new treatment (identified by a specific fee position) and encompassed all sessions provided by a physiotherapist following a patient referral by a physician. To count as an episode, the gap between consecutive visits had to be less than six months and no new treatment had been undertaken. The unit of observation was thus the specific encounter between the patient and the physician/physiotherapist. To eliminate incomplete observations with potentially truncated information on the number of sessions, we excluded episodes that involved appointments between 1 July and 31 December 2006. Figure 2 clarifies the criteria we used to delineate the episodes of physical therapy care that we were to study. All studied episodes (numbered 1, 2 and 6.2 in Figure 2) began in 2005 (January 1 to December 31, 2005), involved follow-ups of two years, and featured no sessions during the second semester of 2006. We excluded episodes beginning in 2006 (numbered 5 in Figure 2; not part of the studied population), without follow-up in 2006 (numbered 4 in Figure 2; possibly right truncated) or with a last session during the second semester of 2006 (numbered 3; possibly right truncated because the episode may have involved additional sessions in 2007). We also excluded episodes that did not entail a first session (episode 6.1, left truncated). Patients might have multiple episodes (episodes 7.1 and 7.2), if a specific fee marked the beginning of a new episode (allowed for a condition involving a new body site whatever time elapsed between two consecutive visits to the same physiotherapist), or if the patient switched to another physiotherapist or was referred by another physician.

#### Independent variables

We characterized patient needs by age, gender, nature of the main condition and co-morbidity burden.

As no information on reasons for seeking ambulatory care is routinely collected in Switzerland, we derived clinical conditions for physical therapy referral from three sources: inpatient diagnoses and procedures, dispensed outpatient drugs and the specialization of the referring physician. Inpatient diagnoses (International Classification of Diseases, 10th Revision: ICD-10 codes) and procedures (International Classification of Diseases, 9th Revision, Clinical Modification: ICD-9-CM codes) were linked to insurers’ data via the anonymous linkage code procedure of the Federal Statistical Office (FSO); only a sequential number was delivered [26]. In 2005 and 2006, pharmacists systematically sent drug codes (called pharmacodes) and dispensation dates to insurers for billing purposes.

We used SQLape® grouper [27], which is suited to the nomenclatures used in Switzerland (adaptation of ICD-10 diagnostic codes and ICD-9-CM procedures codes, Anatomical Therapeutical Chemical ATC classification codes from transcoding pharmacodes) to allocate patients to one of the mutually exclusive morbidity groups described in Additional file 1. Hospital diagnoses and interventions, as well as drug-inferred illnesses were retained if they occurred concurrently with a physical therapy episode. Where clinical information was unavailable, we allocated patients based on the specialization of the referring physician (e.g. orthopedics, psychiatry, neurology, surgery or rheumatology). If a patient was assigned to several groups, we retained only one according to a hierarchical categorization (see Additional file 1).

We estimated the co-morbidities burden by means of the cumulated cost of care services in 2005, exclusive of physiotherapist costs, based on the hypothesis that higher medical costs reflect more co-morbid conditions or poorer overall health status.

We characterized physicians and physiotherapists by their anonymous identifiers and by the variation coefficients of the number of sessions per episode. We hypothesized that a larger variation coefficient might reflect a greater propensity on the part of the therapist to respond to the wishes of the patient (responsiveness in Figure 1).

Swiss funding regulations are based on three measures: deductibles from patient out- of-pocket expenses (to discourage discretionary care consumption), the nine-session cap for prescriptions (to limit use for acute conditions and encourage the regular re-evaluation of health benefits for chronic ailments), and a bonus for new treatments (to incentivize physiotherapists to treat new patients rather than extend the treatment of existing patients). The expected relationship between the propensity of a provider to use a set of nine sessions and the volume of physical therapy was not clear-cut, due to the fact that the cap on treatment episodes can both curb and encourage overuse. A high proportion of new treatments per provider was expected to shrink the volume of physical therapy expenditure per treatment episode.

Context variables may also modify the outcome, if there are several physicians or physiotherapists involved in the treatment of one condition, leading to a spurious splitting of episodes. For instance, these biases were caused by the use of locum physiotherapists to provide holiday cover, the decision of the patient to continue their treatment with a physiotherapist closer to where they lived or the renewal of treatment made by another physician.

To overcome geographical differences in provider densities, we considered the canton where patients lived and whether they lived in an urban setting. The FSO classifies residency at four levels, the most urban being one of the 62 large Swiss towns [28].

### Statistical analysis

**Figure 3:**
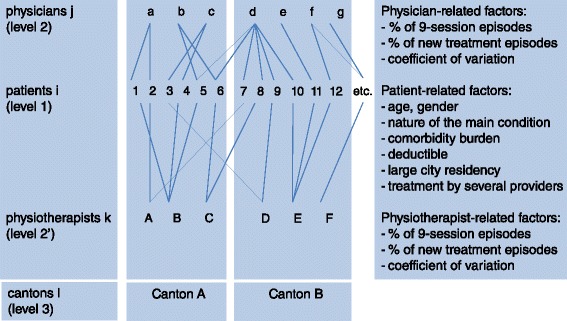
**Hierarchical and cross-classified data structure.**

The data had a hierarchical and cross-classified structure, with patients (level 1) nested within the cells of the cross-classification of physicians and physiotherapists (both at level 2, as physicians had ongoing relationships with several physiotherapists, and physiotherapists with multiple physicians, see Figure 3). Physicians and physiotherapists were nested within cantons (level 3). We therefore used a multilevel regression model with crossed random effects for physiotherapists and physicians and with fixed effects for cantons [29,30]. We assessed potentially explanatory variables at their various levels (see Figure 3). One quarter of patients had multiple episodes over time; adjusting for these correlations would dramatically increase modeling complexity [31]. We therefore restricted our analyses to the first episode per patient. Although this strategy did not use all the information contained in the data, it remained effective thanks to the large sample size and the time-invariant explanatory factors.

The dependent variable was the number of sessions per physical therapy episode. It is in principle a count variable. However, as number of sessions varied from 1 to 330 and the distribution was highly right-skewed, the variable was log-transformed. This allowed to nicely symmetrize the distribution of the outcome (thereby defining a log-linear or semi-log model), making it appropriate for analysis by multilevel methods for linear models. This was of great advantage given the well-known difficulty in reliably estimating such complex multilevel generalized linear models for count data [32]. Another advantage of our approach is that the order two moment was independent from the order one moment, which is not the case with the more constrained and less general Poisson or Negative Binomial models. We, therefore, analyzed the log-dependent variable by methods for multilevel-cross-classified linear models [33]. In a semi-log model, the regression coefficient associated with a particular variable estimates the semi-elasticity, i.e. the percentage change (in linear approximation), of the number of sessions for a unit change of the independent variable (log(*y*) = *β*_0_ + *β*_1_*x* + *ε* and *d* log(*y*)/*dx* = (*dy*/*y*)/*dx* = *β*_1_).

The unconditional model used to partition the overall variance across the levels is written in Additional file 2, part 1. The conditional model used to quantify the contribution of patient, physician and physiotherapist characteristics to explaining the outcome variance is written in Additional file 2, part 2.

The total unexplained variance in the outcome variable was partitioned into the three variance components: patient (level 1), physician (level 2), and physiotherapist (level 2’) by the appropriate intra-class correlation coefficient (ICC) [34]. The proportion of variation (PEV) at each level that was explained by the various factors, as well as the overall explained variance were computed using the unconditional and conditional estimated residual variances, which is akin to computing the extra contribution to the model R^2^ when the additional set of predictors is included in the regression model (i.e. the square of the multiple semi-partial correlation coefficient) [34,35]. Unless the regressors are all orthogonal, the factor-specific PEVs do not add up to the total PEV, the difference representing the collinearity effect due to the inclusion of all regressors in the model [36].

To assess the impact of the three “manageable” variables (deductibles, proportion of nine-session sets and new treatments) on the number of sessions, we used a back-transformation to compute the predictions on the original scale [37]. Note that the semi-elasticity allows us to compute the percentage change, whereas the back-transformation approach makes it possible to calculate the absolute difference in the number of sessions provided.

To assess the impact of the most and least parsimonious practices, we computed empirical Bayes estimates of the random effects for both physicians and physiotherapists. We also calculated the difference in the number of sessions between providers at the 2.5 and 97.5 percentiles of the distribution [34].

Using SAS 9.3, we estimated the regression models by maximum likelihood and assessed the goodness of fit using scatter plots of marginal and conditional residuals versus fitted values.

## Results

We identified 122,841 patients who had received physical therapy during 2005. The total number of treatment episodes was 169,305. These were dispensed by 4,029 physiotherapists based on referrals by 9,476 physicians. The decision to include only the patients’ first episode and to exclude physicians and physiotherapists with only one observation (missing coefficient of variation) led to a studied population of 87,866 episodes dispensed by 3,365 physiotherapists based on referrals by 6,131 physicians.

Table 1 compares the source and studied population. The latter exhibited a weaker severity: younger subjects, fewer conditions assigned to a clearly defined musculoskeletal condition (16% vs 26%), weaker co-morbidity burden, and less frequent incidence of treatment management by several care providers (more than 95% were treated by a single physician and physiotherapist versus 58% and 69% respectively in the source population). In contrast, physician- and physiotherapist-related variables were similar in the two samples.

**Table 1:** **Mean volume (number of physical therapy sessions per treatment episode) according to patient, physiotherapist and referring physician characteristics in the source and studied population**

	**Source population**		**Studied population** ^**a**^	
	**N (%) or mean value of the category**	**Mean volume (SD)**	**N (%) or mean value of the category**	**Mean volume (SD)**
**Variables**	**N = 169,305**	**12.1 (11.9)**	**N = 87,866**	**11.7 (10.8)**
**Patient factors**				
Age (years)				
<18	10,667 (6%)	8.9 (8.3)	6,928 (8%)	8.5 (7.0)
19-39	30,998 (18%)	10.3 (9.5)	18,546 (21%)	9.8 (8.0)
40-59	59,987 (35%)	12.1 (11.4)	31,150 (35%)	11.7 (10.1)
60-79	52,318 (31%)	13.1 (12.6)	24,241 (28%)	13.0 (11.9)
80+	15,335 (9%)	14.8 (16.4)	7,001 (8%)	14.8 (16.0)
Gender				
Male	62,869 (37%)	11.8 (11.9)	35,472 (40%)	11.3 (10.6)
Female	106,436 (63%)	12.3 (12.0)	52,394 (60%)	11.9 (10.9)
Nature of the main condition				
Neurological disease	6,086 (4%)	17.5 (22.2)	2,336 (3%)	16.9 (21.4)
Shoulder surgery^b^	1,722 (1%)	16.9 (14.4)	337 (0%)	19.0 (15.8)
Muscle disease^b^	1,112 (1%)	14.8 (14.4)	331 (0%)	15.3 (14.7)
Other hip surgery^b^	1,827 (1%)	14.6 (13.3)	489 (1%)	13.9 (12.3)
Knee prosthesis^b^	3,363 (2%)	14.4 (12.7)	661 (1%)	16.9 (13.7)
Rehabilitation	863 (1%)	14.4 (17.0)	285 (0%)	12.5 (11.2)
Hip prosthesis^b^	4,553 (3%)	13.6 (12.3)	848 (1%)	13.9 (12.2)
Other orthopedics^b^	3,055 (2%)	13.6 (13.1)	1,629 (2%)	13.4 (14.4)
Back^b^	8,563 (5%)	13.5 (13.6)	2,388 (3%)	13.6 (14.3)
Osteoporosis^b^	5,701 (3%)	13.4 (13.2)	2,474 (3%)	13.6 (12.2)
Cancer	3,178 (2%)	13.1 (13.4)	1,421 (2%)	13.2 (14.2)
Trauma^b^	4,947 (3%)	13.0 (13.6)	2,111 (2%)	12.5 (12.1)
Lymphatic and breast disease	1,587 (1%)	13.0 (12.9)	600 (1%)	12.4 (11.4)
Knee^b^	469 (0%)	12.6 (9.2)	173 (0%)	13.4 (10.4)
Rheumatology^b^	7,887 (5%)	12.3 (11.7)	3675 (4%)	12.4 (11.3)
Psychiatry	34,242 (20%)	12.3 (11.8)	17,588 (20%)	12.3 (11.5)
Other (medical)	41,592 (25%)	11.0 (9.7)	25,764 (29%)	10.9 (9.0)
Surgery	5,529 (3%)	10.8 (9.0)	3,203 (4%)	10.3 (7.6)
Any of above	33,029 (20%)	10.7 (9.7)	21,553 (25%)	10.4 (8.3)
Comorbidity burden (annual health care costs, mean in 10^3^ CHF)				
<1000	27,242 (16%)	10.3 (8.8)	22,319 (25%)	10.1 (8.1)
1001-2000	20,062 (12%)	10.5 (8.3)	13,754 (16%)	10.4 (7.9)
2001-5000	44,321 (26%)	11.2 (8.9)	24,725 (28%)	11.3 (8.6)
5001-10000	36,000 (21%)	12.2 (10.6)	14,805 (17%)	12.5 (10.5)
10001-20000	24,169 (14%)	13.4 (13.0)	7,451 (8%)	14.1 (13.8)
20001-50000	13,626 (8%)	16.1 (18.5)	3,747 (4%)	16.8 (19.2)
>50000	3,885 (2%)	22.3 (30.1)	1,065 (1%)	24.5 (32.8)
Deductibles				
<=300	98,499 (58%)	12.4 (12.6)	50764 (58%)	11.9 (11.4)
301-600	55,734 (33%)	12.2 (11.3)	27582 (31%)	11.7 (10.4)
601-1500	11,984 (7%)	10.4 (8.6)	7484 (9%)	10.1 (7.5)
>1500	3,088 (2%)	10.0 (9.5)	2036 (2%)	9.6 (8.3)
Large city residence^c^				
No	104,960 (62%)	12.0 (11.8)	55680 (63%)	11.5 (10.7)
Yes	64,345 (38%)	12.4 (12.0)	32186 (37%)	11.9 (10.9)
Number of physicians per patient				
1	98,864 (58%)	11.7 (10.9)	84231 (96%)	11.6 (10.6)
2	45,346 (27%)	12.6 (12.9)	3633 (4%)	12.6 (13.6)
>2	25,095 (15%)	13.1 (13.7)	2 (0%)	5.5 (3.5)
Number of physiotherapists per patient				
1	116,868 (69%)	12.1 (11.7)	85332(97%)	11.6 (10.7)
2	39,471 (23%)	12.3 (12.3)	2426 (3%)	12.8 (11.8)
*>2*	12,966 (8%)	12.0 (12.4)	108 (0%)	13.4 (12.7)
**Physician factors**				
Proportion of treatments by physician as a nine-session episode by quintiles rank				
1^st^	22%	10.5 (11.8)	21%	9.8 (10.1)
2^nd^	49%	11.1 (11.2)	48%	10.6 (10.3)
3^rd^	63%	12.4 (12.5)	63%	11.9 (11.3)
4^th^	73%	13.3 (12.0)	73%	12.8 (11.0)
5^th^	86%	13.4 (11.9)	85%	13.2 (10.7)
Proportion of new treatments by physician by quintiles rank				
1^st^	48%	15.2 (16.8)	50%	14.3 (15.2)
2^nd^	60%	12.7 (12.0)	61%	12.0 (10.6)
3^rd^	65%	11.7 (10.5)	66%	11.5 (9.9)
4^th^	71%	11.3 (9.8)	72%	10.9 (8.7)
5^th^	81%	9.8 (7.6)	81%	9.6 (7.1)
Variation coefficient of the physician (by quintiles rank)^d^				
1^st^	0.45	10.3 (5.8)	0.38	9.9 (4.7)
2^nd^	0.65	11.4 (7.8)	0.56	10.8 (6.7)
3^rd^	0.76	11.6 (9.4)	0.67	11.6 (8.3)
4^th^	0.88	13.0 (12.1)	0.80	11.9 (10.1)
5^th^	1.19	14.5 (19.3)	1.14	14.1 (18.2)
**Physiotherapist factors**				
Proportion of treatments by physiotherapist as a nine-session episode by quintiles rank				
1^st^	29%	11.0 (12.6)	30%	10.2 (11.2)
2^nd^	46%	11.2 (11.1)	47%	10.8 (10.4)
3^rd^	56%	12.0 (11.4)	56%	11.4 (10.0)
4^th^	68%	12.8 (12.3)	68%	12.4 (10.7)
5^th^	84%	13.8 (11.9)	84%	13.5 (11.1)
Proportion of new treatments by physiotherapist by quintiles rank				
1^st^	49%	14.9 (16.9)	51%	14.2 (15.0)
2^nd^	60%	12.6 (11.7)	61%	12.0 (10.5)
3^rd^	66%	11.8 (10.8)	51%	14.2 (10.0)
4^th^	71%	11.0 (9.4)	61%	12.0 (8.6)
5^th^	80%	10.3 (8.4)	67%	11.5 (7.7)
Variation coefficient of the physiotherapist (by quintiles rank)^e^				
1^st^	0.53	10.8 (6.4)	0.46	10.4 (5.6)
2^nd^	0.68	11.4 (8.2)	0.61	10.7 (7.0)
3^rd^	0.78	11.7 (9.6)	0.70	11.4 (8.4)
4^th^	0.89	12.5 (11.8)	0.81	11.7 (9.9)
5^th^	1.20	14.3 (19.1)	1.13	14.1 (18.0)

^a^Restricted to the first treatment episode per patient and patients treated by a physiotherapist and a physician with at least two observations (allowing a value of the coefficient of variation).

^b^Referred to as musculoskeletal conditions in the manuscript.

^c^In one of the 62 largest cities.

^d^N = 167,196 because of missing values of the coefficient of variation.

^e^N = 168,979 because of missing values of the coefficient of variation.

The median number of sessions per episode was nine (interquartile range 6–13); the distribution had a long right tail with a maximum of 330. Such cases, which reflect the existence of chronic illness management, for instance provision of respiratory support, substantiate our choice of applying the log-linear modelling of data. In bivariate analyses, higher use was associated with older age, women, higher annual health care costs and specific conditions. We observed the highest use for neurological diseases and shoulder operations, while use was lowest for unspecific medical conditions and non-orthopedic surgery. Higher deductibles were associated with lower use. More sessions were associated with a higher proportion of nine-session episodes and a lower proportion of new treatments, computed by physician or physiotherapist. A large range of variation coefficient of the physiotherapist or the physician was associated with higher use.

The results of the multivariate analysis are shown in Table 2. We found a significant link between all of the above predictors and the number of sessions.

**Table 2:** **Multilevel regression analysis of the number of physiotherapy session** ^**a**^

	**Model coefficients** ^**c**^	
**Factors** ^**b**^	**Estimates (i.e. semi-elasticities)**	**95% confidence interval**
***Patient needs***		
Age and gender		
[20-39] men	.088	.062- .114
[40–59] men	.217	.193- .242
[60–79] men	.260	.235- .285
[80 + ] men	.211	.175- .247
[20-39] women	.095	.071- .120
[40–59] women	.232	.208- .255
[60–79] women	.291	.266- .315
[80 + ] women	.259	.230- .288
Conditions^d^		
Shoulder operation	.324	.257- .391
Total knee prosthesis	.252	.203- .301
Muscular disease	.175	.107- .242
Neurological diseases	.081	.053- .109
Other orthopedic surgery	.043	.011- .074
Osteoporosis	.035	.008- .062
Rheumatic conditions	.024	.002- .047
Other surgery	-.043	-.067- -.019
Lymphatic problems	-.055	-.106- -.004
Rehabilitation	-.084	-.157- -.011
Comorbidity burden (annual costs 10^3^ CHF)		
1,001–2,000	.020	.006- .033
2,001–5,000	.065	.053- .077
5,001–10,000	.130	.115- .144
10,001–20,000	.180	.162- .198
20,001–50,000	.223	.199- .246
>50,000	.381	.340- .421
***Provider practices***		
Variation coefficient of the physician (by quintiles rank)		
2^nd^	.043	.028- .058
3^rd^	.062	.046- .078
4^th^	.062	.046- .078
5^th^	.082	.065- .099
Variation coefficient of the physiotherapist (by quintiles rank)		
2^nd^	.019	.000- .038
3^rd^	.037	.018- .057
4^th^	.065	.044- .086
5^th^	.087	.067- .107
***Funding regulations***		
Deductibles (CHF)		
601–1,500	-.060	-.076- -.044
>1,500	-.086	-.115- -.058
Ceiling per prescription (proportion of treatments by physician as a nine-session episode by quintiles rank)		
2^nd^	.080	.064-.097
3^rd^	.154	.137- .171
4^th^	.205	.188- .223
5^th^	.235	.216- .254
Ceiling per prescription (proportion of treatment by physiotherapist as a nine-session episode by quintiles rank		
2^nd^	.081	.059- .103
3^rd^	.114	.092- .136
4^th^	.155	.132- .177
5^th^	.227	.202- .252
Proportion of new treatments by physician by quintiles rank		
2^nd^	-.050	-.066- -.033
3^rd^	-.058	-.074- -.041
4^th^	-.095	-.112- -.078
5^th^	-.154	-.171- -.137
Proportion of new treatments by physiotherapist by quintiles rank		
2^nd^	−0.045	-.064- -.025
3^rd^	−0.071	-.091- -.051
4^th^	−0.081	-.101- -.060
5^th^	−0.104	-.125- -.082
***Context variables*** ^***e***^		
Being treated by more than one physician	-.083	-.122- -.045
Being treated by more than one physiotherapist	.060	.015- .105
Residency canton (13 dummy variables)		
Only one had a significant coefficient	.042	.006-.078
***Model constant***	1.732	1.688-1.776

^a^Analysis was restricted to the patients’ first treatment.

^b^Only variables with a coefficient significantly different from 0 (p < 0.05) are shown.

^c^The model coefficients can be interpreted as semi-elasticities, i.e. the percentage change of the outcome after a unit change of the explanatory variable. The reference category was: men, < 20 y, no condition identified from drugs prescriptions, inpatient diagnoses or a specialist contact, deductibles < CHF 600, being treated by one physician and one physiotherapist in the lowest quintiles rank for all their variables, and not resident in a large city.

^d^The following conditions had no significant effect: cancer, mental conditions, back problems, other knee problems, total hip prosthesis, other hip surgery, other trauma, and other medical condition.

^e^Urban residence had no significant effect.

Physical therapy use increased with age until 79 years; a 70-year-old man had a 4.3% higher use than a 50-year-old man (as shown by the difference between the two semi-elasticities, i.e. .260-.217). Use was slightly higher among women regardless of age (about 2% on average). Patients with the highest health care spending had almost 40% more sessions than people who spent the least. Shoulder surgery accounted for a 32.4% increase. Patients with the highest deductible had on average 8.6% fewer visits than those with the lowest deductibles.

The higher proportion of nine-session episodes was associated with higher use: switching from the lowest to the highest proportion increased use by 23% for both physicians and physiotherapists. A higher proportion of new treatments had an opposite effect: use shrank by more than 10% for physiotherapists and by more than 15% for physicians. A larger coefficient of variation, i.e. better responsiveness, was associated with higher use (about 8% for both physicians and physiotherapists).

We found no significant link between the number of sessions and the place of residence (urban or not) and cantonal indicators (except one). Being treated by several physicians lowered use, whereas being treated by several physiotherapists increased use.

Estimates of variance components are shown in Table 3. Most of the variation in the number of sessions was due to differences between patients (88.8% of the total variance) and not between physicians (4.9%) or physiotherapists (6.3%). The overall variance explained by the full model was rather weak (11.2%) despite the large coefficients of most factors.

**Table 3:** **Proportion of variation explained by grouping structure and covariates**

			**Variance explained in % (PEV)** ^**a**^							
					**Funding regulations**					
	**Variance** ^**b**^	**ICC** ^**c**^	**Context**	**Health factors**	**Deductibles**	**% New treatments**	**% Nine sessions series**	**Responsiveness**	**Collinearity** ^**d**^	**Total**
**By grouping level** ^**e**^										
Physician	.021	.049	.000	.079	.000	.048	.251	.029	.323	.730
Physio.	.028	.063	.022	.033	.000	.055	.174	.032	.274	.590
Patient	.393	.888	.000	.040	.001	.002	.000	.000	.002	.045
**Overall**	.442	1								.112

^**a**^The proportion of explained variation (PEV, i.e. squared semi-partial correlation coefficient) represents the amount of variance that is explained by the regressors included in the model.

^**b**^Total variance potentially explained at all levels.

^**c**^The intra-class correlation coefficient (ICC) allows the partitioning of the total variability in the outcome into its three variance components: physicians, physiotherapists and patients.

^**d**^Unless the regressors are all orthogonal, the prognostic factors’ specific PEVs do not add up to the total PEV, the difference representing the collinearity effect due to the inclusion of all regressors into the model.

^**e**^The third level (canton) was treated as a fixed effect and therefore no variance component appears in the disaggregation of the total variance.

Our prognostic factors explained a large proportion of variance between physicians and between physiotherapists: 73% and 59%, respectively. For physicians, pricing rules explained the largest proportion: 25% for the proportion of nine-session sets, and 5% for the proportion of new treatments. For physiotherapists, it was 17% and 6%, respectively. Health status was an important explanatory factor for variations between physicians (8%). Responsiveness, health factors and context (management of condition by several physiotherapists) had a similar predictive ability on variations between physiotherapists (2 to 3%).

At 4.5%, the proportion of explained variance between patients was very low. The most powerful prognostic factor was health status.

Using the back-transformation approach, we found that switching from the lowest (<600 Swiss francs) to the next highest deductible (601–1,500 Swiss francs) would save an average of 0.7 sessions per treatment episode. Likewise, restricting the proportion of nine-session episodes to the fourth quintile, i.e. maximum of 73% for physicians and maximum of 68% for physiotherapists (upper limit of the 4^th^ quintile) would save a total of 1.4 sessions. Increasing the proportion of new treatments above the first quintile, i.e. over 60% (upper limit of the first quintiles for both care providers), would save a total of 1.1 sessions.

The differences between the least and most parsimonious practices were 1.3 sessions per treatment for physicians and 2.7 for physiotherapists.

## Discussion

The intensity of physical therapy utilization was relatively high in our population compared to other countries, which would suggest that effectiveness in Switzerland is sub-optimal. In a national non-selected sample of US adults, the average number of visits per episode was 9.6 [13], whereas an older population of Medicare beneficiaries attended a mean of 6.8 appointments for musculoskeletal conditions [38]. In the Netherlands, the treatment of similar conditions required an average of 10.5 visits [39]. A cross-country comparison found substantial variation in the type of treatment given and the number of visits per episode [2]. In this study, corrected for age, gender and episode duration, mean numbers were 10.0 in the US, 6.5 in Israel and 10.0 in the Netherlands.

Our study confirms that the considerable variation in the intensity of physical therapy per treatment episode depends on both health-related and non-health related factors.

Poorer health status, reflected by higher health care costs, was associated with a higher number of visits. A higher number of sessions among women was consistent with their usual higher level of care use [40]. The gender difference might also be related to non-measured morbidity, such as levels of pain or impairment, which tend to be more severe for women with musculoskeletal complaints than for their male counterparts [41]. Like other studies, we found that shoulder and knee impairments were associated with more visits [38] and that having surgery also increased the number of visits [42]. The decline in use among the oldest age groups has also been found by other studies [43,44].

Insurance status was consistently shown to affect the use of physical therapy services [43,44]; higher deductibles decreased utilization, suggesting underuse among patients who chose the highest out-of-pocket payment option.

More physical therapy sessions were associated with physicians and physiotherapists with the best responsiveness (i.e. highest coefficients of variation).

The higher proportion of nine-session sets was associated with more intense use of physical therapy, indicating that prescription caps did not hinder the number of sessions per episode. Restricting the number of sessions has been the standard payment policy to limit overuse. Our findings, however, challenge its effectiveness. In Israel, where there is no cap but rather long waiting lists, patients with acute complaints receive more sessions than those with chronic complaints, probably because the expected improvement is better among the former group [2]. In Switzerland, the ceiling is set at nine sessions, corresponding to the median number of sessions. This cap might have no moderating effect on the many patients requiring less than nine sessions and may, in fact, encourage the provision of 18 sessions to patients who would require slightly more than nine sessions. We recommend a lower ceiling for the majority of acute conditions, as the literature and our own data would suggest that six sessions per prescription in non-surgery conditions is adequate [2,17,45].

Physiotherapists with a higher rate of new treatments tended to provide fewer sessions than others. This might indicate that physiotherapists tend to compensate for the smaller volume of new patients by extending the treatment of existing patients. However, we found the same association for physicians (more new physical therapy referrals was associated with lower intensity of physical therapy use), even though they have no financial incentives. This finding may also reflect differences in the population served: patients with chronic complaints generally received more sessions than those with acute ailments, provided there is not an undersupply of physiotherapists [17].

Contrary to several studies elsewhere, we did not find significant variations across urban and rural residency and across geographical areas, which would indicate that there was no overt rationing of physical therapy in Switzerland. However, more homogenous health services area than cantons on factors that determine provision and utilization of health resources might yield different findings.

The proportion of explained variation (PEV) by physician and physiotherapist characteristics represented only a small component of total variance (5% and 6% respectively). The factors introduced in the model largely explained more than half of the associated PEV (levels 2 and 2’), thereby illustrating that unobserved provider-related variables would not improve the model significantly. Funding rules in Switzerland, especially caps, were the most important determinants. Health factors accounted for a larger proportion of variation among physicians (7.9%) than among physiotherapists (3.3%). This is not surprising given that the two health care providers base their decisions on different models: physicians use the biomedical model which is based on diagnosis, while physiotherapists apply the biopsychosocial model which is based on functional deficit [46].

These findings suggest that providers tend to maximize the benefits offered by the regulations (not necessarily for their own benefit but rather for ensuring patient satisfaction). Not only does this highlight the need to review the current nine-session ceiling but it also argues in favor of further studies that explore financial incentives with the achievement of better clinical outcomes [45].

Episodes with multiple medical referrals were associated with fewer sessions, probably reflecting a fractioning of episodes. The inverse relation observed for physiotherapists might reflect complex and chronic conditions requiring the involvement of several physiotherapists, or the fact that a colleague replaces the attendant physiotherapist in their absence, which is a common occurrence. In fact, our case-mix measure took insufficient account of the duration of complaints, which has been consistently associated with higher use [2,12,17].

Of the total unconditional variance, the largest share was attributable at the patient level (89%). However, patient characteristics, such as health conditions, explained only 4% of this variance (Table 3), suggesting that the main determinants of variability were unmeasured.

This unexplained large variation in utilization has been found in other studies, even in those which encompass more detailed clinical information or consider only specific conditions [38]. This degree of variation, which does not appear to be attributable to illness severity or disease characteristics, is generally viewed as a potential threat to overall quality of care. However, there must be many unmeasured factors over which the provider has little control like psychosocial variables, including pain behavior, negative beliefs or coping style. Functional status is certainly also of interest but not routinely recorded. Inappropriate factors like patient requests that yield no benefits might also be part of the unexplained variation. Studies have shown that physicians’ clinical decision-making is heavily influenced by patients’ persistent requests for various services, which themselves depend more on subjective health complaints than on objective morbidity burden as measured by a count of chronic conditions [47]. Providers might also be reluctant to restrict the provision of physical therapy services when they do not have a satisfactory alternative to offer their patients. Finally, medical intervention can have psychologically mediated benefits, even when medically unnecessary. As a result, it would be probably insufficient to undertake basic efficiency monitoring according to the expected number of required sessions. Monitoring of chronic high users should be based on a review of the treatment plan according to the following criteria: expected number of sessions to reach defined goals, compliance with the treatment plan and improvement of functional outcomes [45].

Data on the relationship between the intensity of physical therapy use and outcomes are scarce and contradictory. According to several studies, attending more sessions was associated with an improvement in most functional outcomes [38]. However, poorer outcomes associated with more visits among patients suffering from back problems suggested a tendency among therapists to add appointments when outcomes did not improve [48]. For neck pain, standard physical therapy may be only marginally better than a brief physical therapy intervention which encourages self-management [49].

### Limitations

The main limitation of the study is its reliance on routinely available data. Several important utilization drivers at the patient level were missing, such as functional disability, behavioral factors and symptom severity. Considering that variance in the number of sessions is found predominantly at the patient level, additional research should focus on the contribution of these factors (possibly also using qualitative research methods) to devise strategies to improve the adequacy of physiotherapy use. Given that psychosocial factors, such as catastrophizing, fear avoidance, and poor coping skills, are known to be important predictors of outcome among musculoskeletal conditions, interventions that deal with patients’ beliefs and attitudes might therefore help to hasten recovery [49]. This is in line with the increasing support among physiotherapists of the biopsychosocial model of care [50]. Finally, as no information was available on patient satisfaction or outcomes, we cannot conclude from the study that opportunities exist to reduce physical therapy use.

A second limitation concerns the studied population. Theoretically, there are three possible sources of bias: 1) during the selection of the studied population (exclusion of patients who left their insurance company in 2006); 2) in selecting first episodes (multiple episodes); 3) exclusion of episodes with a final session during the second semester of 2006. We believe that there are several reasons why such bias had no significant impact on our results. First, only 17% of insured left their insurance company in 2006. Secondly, the large size of the sample and the comparisons between the source and studied population support the representativeness of the data in terms of physical therapy practices for the whole country. Thirdly, less than 3% of episodes began in 2005 and ended during the second semester of 2006. The exclusion of certain protracted episodes should not alter the analysis of the general behavior of patients, physicians and physiotherapists.

Although the data we used were eight-years-old, there have been no cost management changes in the intervening period, and the utilization patterns found in the study can be reliably generalized to the current situation.

## Conclusion

Against the backdrop of abundant physical therapy supply, financial incentives as applied in Switzerland (nine-session ceiling, higher reimbursement for new treatments) did not restrict utilization. The multilevel regression analysis exhibited no cantonal variations, but found small variations between physicians and physiotherapists, and revealed that most of the variance was at the patient level. This suggests that regulation should focus more on patients and that further research is required to understand the determinants of patient demand and the effectiveness of physical therapy.

## Additional files

Additional file 1:
**Morbidity groups.**
Additional file 2:
**Measures of explained variation.**

## Abbreviations

SFSO: Swiss federal statistical office
ICD-9-CM: International classification of diseases, 9th revision, clinical modification
ICD-10: International classification of diseases, 10th revision
ATC: Anatomical therapeutical chemical
ICC: Intra-class correlation coefficient
PEV: Proportion of explained variation
