# Supplementary material for: The effect of patient, provider and financing regulations on the intensity of ambulatory physical therapy episodes: a multilevel analysis based on routinely available data
Source: BMC Health Serv Res. 2015 Feb 7;15:52. doi: 10.1186/s12913-015-0686-6 (PMC4325958; doi:10.1186/s12913-015-0686-6)
Supplement: Additional file 1: — Morbidity groups. [file 12913_2015_686_MOESM1_ESM.doc]

**Additional file 1 Morbidity groups.**

|  | Information source | | | | |  | |  |
| --- | --- | --- | --- | --- | --- | --- | --- | --- |
|  | Hospital data | | Insurer data | | |  | |  |
| Morbidity groups | Diagnoses  (SQLape® categories) | Surgical procedures (SQLape® categories) | Inferred from dispensed outpatient drugs | Inferred from physician specialization | | Hierarchy | |  |
| Other hip surgery | - | COX* | - | | - | | 1 | |
| Knee prosthesis | - | GEN4 | - | | - | | 2 | |
| Hip prosthesis | - | COX5 | - | | - | | 3 | |
| Shoulder surgery | - | SCA* | - | | - | | 4 | |
| Back diseases | L-dV, L-fV, L-tV, L-zC, N-oR | COL* | - | | - | | 5 | |
| Lymphatic and breast diseases | F-mM | LYM2, MAM* | - | | - | | 6 | |
| Muscle diseases | - | MUS* | - | | - | | 7 | |
| Other orthopedics | - | ART*, DIG*, MAN*, OSS*, PED* | - | | Orthopedists | | 8 | |
| Other knee surgery |  | GEN2, GEN3 | - | | - | | 9 | |
| Neurological diseases | N-bC, N-dC, N-dM, N-dP, N-hC, N-iC, N-iM, N-iS, N-mC, N-oC, N-sC, N-sT, N-zC, N-zM, N-zS | - | Yes | | Neurologists | | 10 | |
| Trauma | L-tB, L-tC, L-tJ, L-tL, L-tZ, R-tT, T-tS, T-tT, Y-tO, Z-tZ, Z-zD, Z-zX | BRA*, CRU* | - | | - | | 11 | |
| Cancer | D-mI, D-mO, D-mR, D-mS, F-mG, F-mO, H-mH, H-mP, O-mO, R-mP, S-mL, S-mM, S-mO, S-mS, T-mS, U-mU, Z-mC, Z-mM, Z-mR, Z-mZ |  | Yes | |  | | 12 | |
| Rehabilitation | Z-rR |  |  | |  | | 13 | |
| Osteoporosis | L-dO |  | Yes | |  | | 14 | |
| Rheumatology | L-dG, L-iG, L-iL, L-iO, L-zM |  | Yes | | Rheuma tologists | | 15 | |
| Psychiatry | P+xS, P-dZ, P-fH, P-tD, P-xA, P-xS, P-zA, P-zZ | - | Yes | | Psychiatrists | | 16 | |
| Surgery | - | All other surgical procedures except obstetrics |  | | Surgeons | | 17 | |
| Medicine | All other categories except new-born | - | Yes | |  | | 18 | |
| Others | New born | Obstetrics | - | |  | | 19 | |

The SQLape® category labels are given on www.sqlape.com
