# Supplementary material for: The effect of patient, provider and financing regulations on the intensity of ambulatory physical therapy episodes: a multilevel analysis based on routinely available data
Source: BMC Health Serv Res. 2015 Feb 7;15:52. doi: 10.1186/s12913-015-0686-6 (PMC4325958; doi:10.1186/s12913-015-0686-6)
Supplement: Additional file 2: — Measures of explained variation. [file 12913_2015_686_MOESM2_ESM.doc]

**Additional file 2 Measures of explained variation.**

1. To partition the overall variance across the levels, we used the following unconditional model:

The outcome $Y_{ijkl}$ in patient *i*, physician *j*, physiotherapist *k*, in canton *l* was modeled as:

$\log(Y_{ijkl})=\pi_{0jkl}+\varepsilon_{ijlk}$ (level 1)

where $\pi_{0jkl}$ is the intercept coefficient at patient level 1, which represents the mean number of sessions for physician *j* and physiotherapist *k*, in canton *l*, and $\varepsilon_{ijlk}$ the random error at patient level 1;

$\pi_{0jkl}=\beta_{000l}+b_{0j0l}+b_{00kl}$ (levels 2 & 2’)

where $\beta_{000l}$ is the intercept coefficient at physician & physiotherapist levels 2 & 2’, and represents the mean number of sessions in canton *l*, $b_{0j0l}$ the random physician effect in canton *l*, and $b_{00kl}$ the random physiotherapist effect in canton *l*;

$\beta_{000l}=\gamma_{0000}+\sum_{l=1}^{L} \gamma_{000l}D_{l}$ (level 3)

where $\gamma_{0000}$ is the grand mean, and $\gamma_{000l}$ is the fixed slope coefficient associated with indicator variable $D_{l}$ for canton *l*;

It is assumed that:

$$\begin{matrix} b_{0j0l}\sim N(0,\sigma_{b_{0j0l}}^{2}) \\ b_{00kl}\sim N(0,\sigma_{b_{00kl}}^{2}) \\ \varepsilon_{ijkl}\sim N(0,\sigma_{\varepsilon}^{2}) \end{matrix}$$

and

$$b_{0j0l}\perp b_{00kl}\perp\varepsilon_{ijkl}\perp\text{all the regressors}$$

1. To quantify the contribution of patient, physician and physiotherapist characteristics with a view to explaining the outcome variance, we estimated the following conditional model:

$\log(Y_{ijkl})=\pi_{0jkl}+\sum_{q=1}^{Q} \pi_{qjkl}(\text{patient factor }q\text{)}_{ijkl}+\varepsilon_{ijlk}$ (level 1)

where $\pi_{qjkl}$ is the slope coefficient at patient level 1 associated with patient factor *q*, for physician *j*, physiotherapist *k*, and canton *l*;

$\begin{matrix} \pi_{0jkl}=\beta_{000l}+\sum_{m=1}^{M} \beta_{0m0l}(\text{physician factor }m\text{)}_{jkl}+b_{0j0l}+ \\ +\sum_{p=1}^{P} \beta_{00pl}(\text{physiotherapist factor }p\text{)}_{jkl}+b_{00kl} \\ \pi_{qjkl}=\beta_{q00l} \end{matrix}$ (levels 2 & 2’)

where $\beta_{0m0l}$ is the slope coefficient at physician level 2 associated with physician factor *m* in canton *l*, $\beta_{00pl}$ the slope coefficient at physiotherapist level 2’ associated with physiotherapist factor *p* in canton *l*, and $\beta_{q00l}$ the intercept coefficient at physician & physiotherapist levels 2 & 2’ associated with patient factor *q* in canton *l*;

$\begin{matrix} \beta_{000l}=\gamma_{0000}+\sum_{l=1}^{L} \gamma_{000l}D_{l} \\ \beta_{0m0l}=\gamma_{0m00} \\ \beta_{00pl}=\gamma_{00p0} \\ \beta_{q00l}=\gamma_{q000} \end{matrix}$ (level 3)

where $\gamma_{0m00}$, $\gamma_{00p0}$, and $\gamma_{q000}$ are fixed intercept coefficients.
